# The efficacy of gasless endoscopic thyroidectomy via trans-subclavian approach in unilateral papillary thyroid carcinoma

**DOI:** 10.3389/fendo.2025.1621481

**Published:** 2025-08-27

**Authors:** Xiangdang Yin, Jingyao Fu, Zhe Zhang, Rui Zhang, Zhaojun Li, Liyou Song, Tong Jin, Xudong Wang

**Affiliations:** ^1^ Department of Head and Neck Oncology, Tianjin Medical University Cancer Institute and Hospital, Key Laboratory of Basic and Translational Medicine on Head & Neck Cancer, Tianjin, China; ^2^ Key Laboratory of Cancer Prevention and Therapy, Tianjin Cancer Institute National Clinical Research Center of Cancer, Tianjin, China; ^3^ Department of Oral-Maxillofacial-Thyroid Oncosurgery, Jilin Cancer Hospital, Changchun, Jilin, China

**Keywords:** endoscopy, trans-subclavian approach, papillary thyroid carcinoma, feasibility, safety

## Abstract

**Objective:**

To evaluate the efficacy of gasless endoscopic thyroidectomy via the trans-subclavian approach in treating unilateral papillary thyroid carcinoma.

**Methods:**

A retrospective analysis was conducted on 140 patients who underwent surgical treatment for unilateral papillary thyroid carcinoma at the Department of Oral-Maxillofacial-Thyroid Oncosurgery, Jilin Cancer Hospital, between February 2023 and August 2024. Patients were divided into the endoscopic (n=70) and open surgery group (n=70). Clinical characteristics, surgical indicators (operation time, number of central lymph node dissections, intraoperative blood loss, 24-hour postoperative drainage volume, indwelling time of drainage tube, surgical cost, length of hospital stay), complication rates, neck discomfort, and incision satisfaction were compared.

**Results:**

Patients in the endoscopic group were younger and had a higher proportion of women (p<0.05). The endoscopic group showed significantly longer operation times and higher 24-hour drainage volumes (p<0.05). No substantial differences were observed between groups in intraoperative blood loss, lymph node dissection count, drainage tube retention time, length of hospital stay, surgical cost, and postoperative complication rates (p>0.05). Neck discomfort was considerably lower, and incision satisfaction was significantly higher in the endoscopic group (p < 0.05).

**Conclusion:**

Gasless endoscopic thyroidectomy via the trans-subclavian approach is a safe and effective treatment for unilateral papillary thyroid carcinoma. It reduces postoperative neck discomfort and improves cosmetic outcomes, making it a viable and promotable surgical option.

## 1 Introduction

Thyroid carcinoma is the most common endocrine malignancy, with its incidence continuing to rise globally (1, 2). Among its subtypes, papillary thyroid carcinoma (PTC) is the most prevalent (3). Surgical intervention remains the primary treatment modality for PTC. However, conventional thyroidectomy via anterior neck incision frequently results in noticeable scarring, which may be unacceptable for patients with high aesthetic expectations. Therefore, endoscopic thyroid surgery has been developed to address this concern. Common endoscopic approaches include trans-chest-breast, trans-axillary, transoral, and postauricular routes (4–6). However, these techniques frequently involve longer operative pathways and extensive flap dissection, resulting in increased hidden trauma. Moreover, some approaches require CO_2_ insufflation, which may lead to complications (7). In recent years, gasless endoscopic thyroidectomy via the trans-subclavian approach has gained popularity in China due to its more discreet incision and shorter operating path. Despite its growing use, limited clinical data are available. This study retrospectively compares the outcomes of gasless endoscopic thyroidectomy via trans-subclavian approach and traditional open thyroidectomy performed at our hospital between February 2023 and August 2024 to evaluate the safety and effectiveness of this minimally invasive technique.

## 2 Materials and methods

### 2.1 Clinical data

This retrospective study included 140 patients with PTC who underwent surgical treatment at the Department of Oral-Maxillofacial-Thyroid Oncosurgery, Jilin Cancer Hospital, between February 2023 and August 2024. All patients underwent unilateral thyroidectomy with isthmusectomy and ipsilateral central neck dissection, performed by the same surgical team. Among them, 70 patients underwent gasless endoscopic thyroidectomy via a trans-subclavian approach (Endoscopic Group), and 70 underwent low cervical arcuate mini-incision surgery (Open Group). Inclusion criteria (1): Preoperative fine-needle aspiration biopsy and postoperative pathology confirmed unilateral PTC with a maximum tumor diameter ≤3 cm and no evidence of lateral neck lymph node metastasis (2); All operations included unilateral thyroidectomy with isthmusectomy and ipsilateral central neck dissection. Exclusion criteria (1): History of neck surgery or radiation (2). Suspected invasion of surrounding tissues, nerves, or blood vessels by the primary or metastatic lesion.

### 2.2 Surgical procedures

Before the operations, patients and their families were informed of the advantages, disadvantages, and potential risks of each surgical approach, and written informed consent was obtained. The same surgical team performed all operations.

#### 2.2.1 Endoscopic group

**Figure 1:**
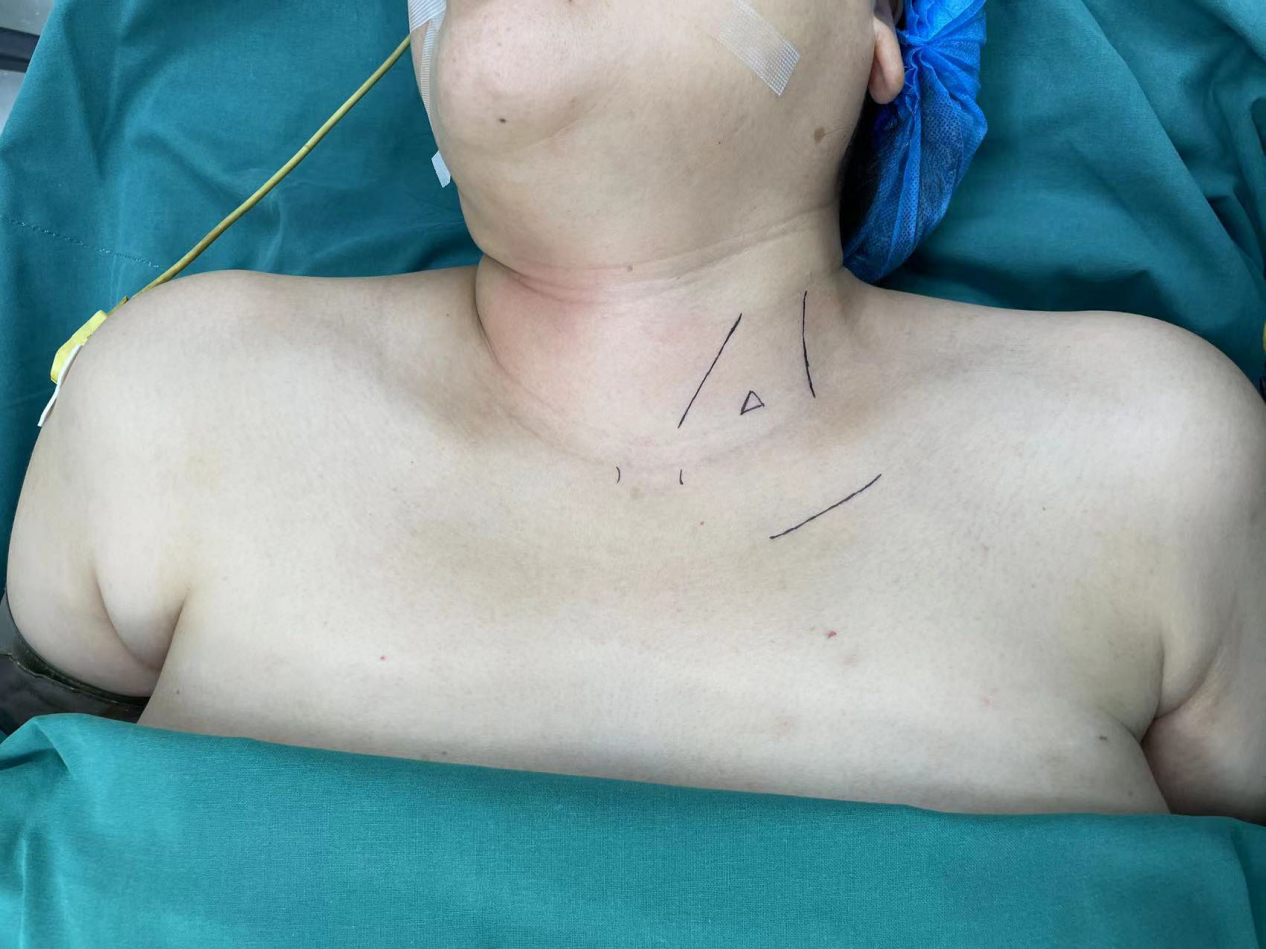
Body surface markers of gasless endoscopic thyroidectomy via trans-subclavian approach.

**Figure 2:**
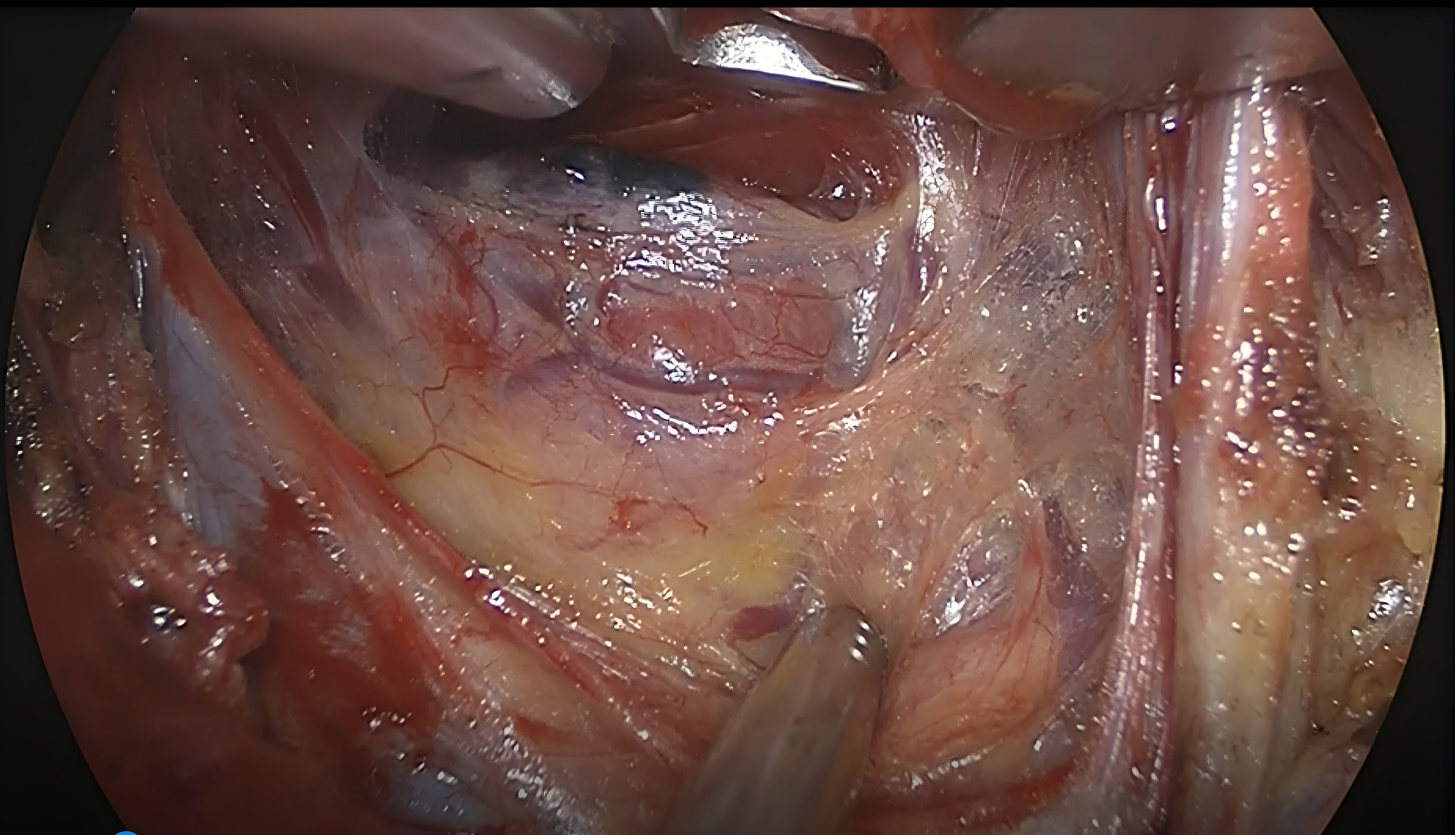
Special retractor suspension exposes the thyroid operative area.

**Figure 3:**
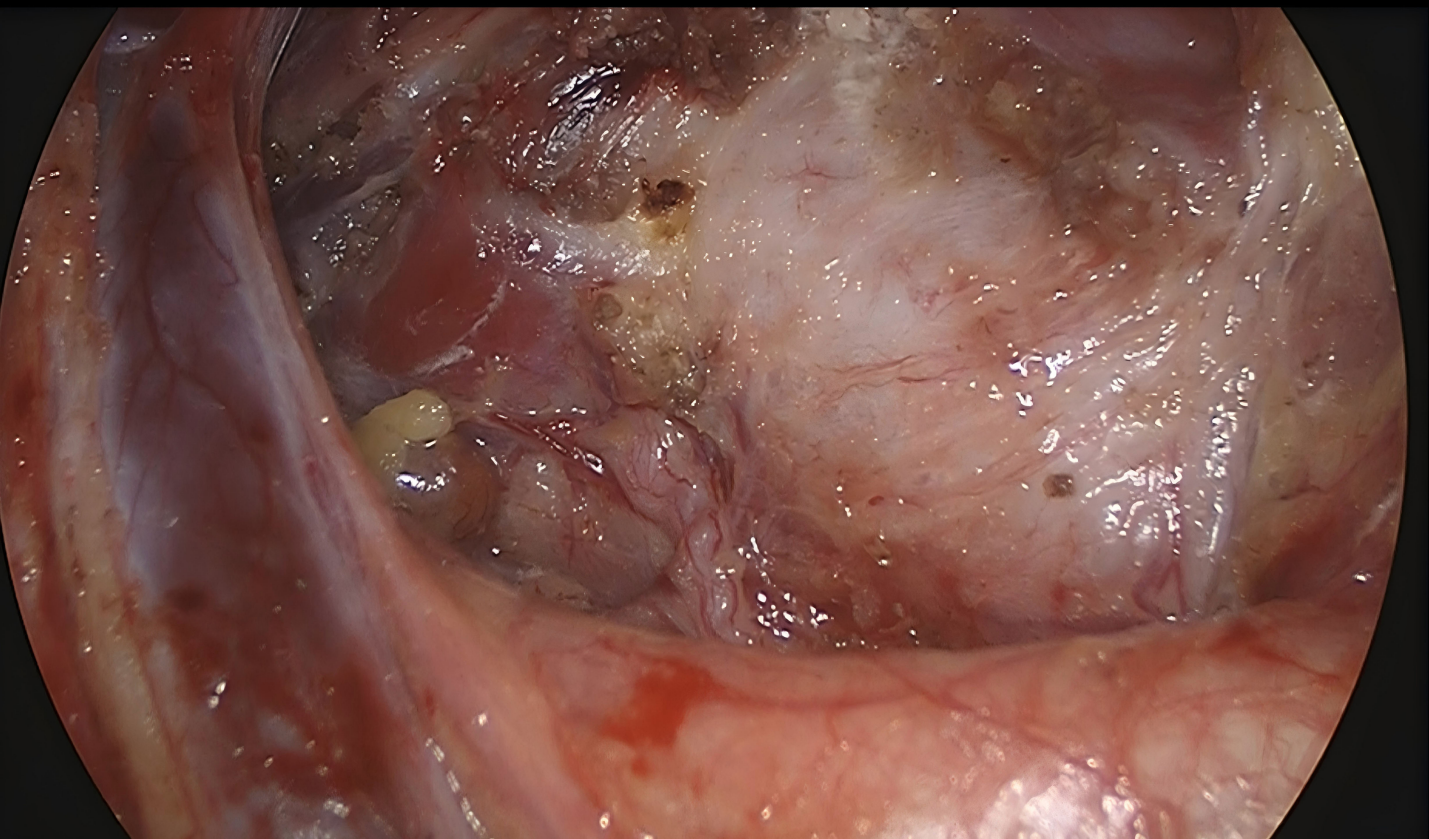
Surgical area after unilateral thyroidectomy with isthmusectomy plus ipsilateral central neck dissection.

Patients were placed in the supine position under general anesthesia with a neuromonitoring endotracheal intubation. A shoulder pad was used to elevate the chest, and the head was extended. The sternal and clavicular heads of the sternocleidomastoid muscle on the affected side were marked. A 3–4 cm surgical incision was made along the natural subclavicular skin fold (**Figure 1**). Following standard disinfection, the skin was incised, and the skin flap was dissected along the fascia of the pectoralis major up to the sternum and supraclavicular region. The natural space between the sternal and clavicular heads of the sternocleidomastoid muscle was exposed, and a suspension retractor was inserted. The carotid sheath was identified, and the sternothyroid muscle was dissected longitudinally along its lateral border toward the midline. The strap muscles and thyroid gland on the affected side were carefully dissected. The retractor was adjusted to suspend and secure the strap and sternocleidomastoid muscles, exposing the thyroid gland and central lymph nodes (**Figure 2**). Nanocarbon was injected into the thyroid gland. The recurrent laryngeal nerve (RLN) was identified in the inferior tracheoesophageal groove using a nerve monitor. The thyroid gland was dissected from inferior to superior and lateral to medial, with careful identification and preservation of the RLN. The inferior thyroid vessels, middle vein, and superior artery and vein were ligated and divided, with attention to preserving parathyroid glands showing negative nanocarbon staining. The affected thyroid lobe, isthmus, and central compartment lymph nodes were resected en bloc. The head was tilted toward the affected side to facilitate the dissection of prelaryngeal fibrofatty tissue (**Figure 3**). The resected specimen was inspected for unintentionally removed parathyroid tissue. The parathyroid glands were immediately autotransplanted into the ipsilateral sternocleidomastoid muscle if found. Hemostasis was ensured, and the surgical field was irrigated with saline. A drainage tube was routinely placed, and the incision was closed in layers, with the skin sutured intradermally using absorbable sutures.

#### 2.2.2 Open group

Patients were positioned supine. Following the induction of general anesthesia, neuromonitoring endotracheal intubation was performed. Shoulders were padded to elevate, and the head was tilted backward. After routine disinfection, a 4-cm arcuate incision was made 2 cm above the sternum. The skin flap was dissected beneath the platysma. The midline of the neck was incised, and the strap muscles were pulled to both sides to expose the thyroid gland. The isthmus was first transected toward the healthy side, and nanocarbon was injected into the affected thyroid lobe. With the assistance of a nerve monitor, the RLN was identified in the tracheoesophageal groove. The thyroid gland was dissected from inferior to superior, with continuous protection of the RLN. The inferior thyroid artery and vein, middle thyroid vein, and superior thyroid vessels were ligated and divided, with careful preservation of negatively stained parathyroid glands. Unilateral thyroidectomy with isthmusectomy was completed on the affected side. Lymphatic and fibrofatty tissues in the prelaryngeal and central compartment (including paratracheal) regions were dissected from medial to lateral. Resected specimens were examined for any unintentionally removed parathyroid tissue. If detected, immediate autotransplantation into the ipsilateral sternocleidomastoid muscle was performed. Hemostasis was confirmed, and the surgical field was irrigated with saline. A drainage tube was routinely placed. The wound was sutured in layers using absorbable protein sutures, with the intradermal skin closure.

### 2.3 Observation indicators

Patient demographic and clinical data were collected, including age, gender, body mass index (BMI), Hashimoto’s thyroiditis status, maximum tumor diameter, multifocality, and tumor location. Surgical parameters included intraoperative time, number of central neck lymph nodes dissected, intraoperative blood loss, 24-hour drainage volume, drainage tube indwelling time, length of hospital stay, and surgical costs. All patients were followed up for 3 months to assess postoperative complications, neck discomfort, and incision satisfaction. Neck discomfort includes symptoms such as a sensation of dysphagia, as well as pain, numbness, pulling sensations, and pressure in the neck. Satisfaction levels were categorized as dissatisfied, fair, satisfied, or very satisfied.

### 2.4 Statistical methods

Statistical analysis was performed using Statistical Package for the Social Sciences (SPSS) statistical software (version 27.0). Measurement data conforming to a normal distribution were expressed as mean ± standard deviation ( $\bar{\text{x}}$ ± s), and the independent two-sample t-test was applied. Measurement data not conforming to a normal distribution were expressed as median (interquartile range) [M(P25, P75)], and the Mann–Whitney U test was used. Count data were expressed as the number of cases, and the chi-square or Fisher’s exact test was applied. Multivariate linear regression analysis was used to adjust for potential confounding variables affecting the operative time and 24-hour postoperative drainage volume of patients. Multivariate logistic regression analysis was used to control for potential confounding variables influencing patients’ neck discomfort and satisfaction with the surgical incision. Statistical significance was set at *P* < 0.05.

## 3 Results

### 3.1 General information

A total of 140 patients participated in this study, with 70 in the endoscopic group and 70 in the open group. The mean age of the endoscopic group (37.57 ± 8.25 years) was significantly younger than that of the open group (46.61 ± 9.49 years), and the endoscopic group had a substantially higher proportion of women (*p*<0.05). However, there were no considerable differences between the two groups in terms of BMI, maximum tumor diameter, multifocality, relative tumor location, and coexisting Hashimoto’s thyroiditis (*p*>0.05) (**Table 1**).

**Table 1:** Comparison of general data between the two groups.

Characteristics	Endoscopic group	Open group	Test Statistic	*P*
Age (year), mean ± SD	37.57 ± 8.25	46.61 ± 9.49	t=6.02	<0.001
Gender (n/%)				
Man	4 (5.7)	18 (25.7)	χ2 = 9.11	0.03
Women	66 (94.3)	52 (74.3)		
BMI (kg/m^2^), median (IQR)	23.32 (21.43, 25.60)	24.71 (22.62, 25.76)	Z=-1.80	0.07
Maximum tumor diameter (cm), median (IQR)	0.50 (0.30, 0.70)	0.50 (0.30, 0.73)	Z=-0.06	0.95
Mutifocality (n/%)				
Single focal	65 (92.9)	58 (82.9)	χ2 = 3.28	0.07
Multifocal	5 (7.1)	12 (17.1)		
Tumor location (n/%)				
Left	29 (41.4)	22 (31.4)	χ2 = 1.51	0.22
Rigt	41 (58.6)	48 (68.6)		
Coexistence of HT (n/%)				
Yes	28 (40)	22 (25)	χ2 = 1.12	0.29
No	42 (60)	48 (45)		

BMI, body mass index; HT, Hashimoto’s thyroiditis.

### 3.2 Surgical-related information

Comparison of surgical outcomes revealed that the endoscopic group had significantly longer operative times and higher 24-hour postoperative drainage volumes than the open group (*p*<0.05). No substantial differences were observed between the two groups in intraoperative blood loss, number of central lymph nodes dissected, indwelling time of drainage tubes, length of hospital stay, and surgical costs (*p*>0.05) (**Table 2**).

**Table 2:** Comparison of surgical-related conditions between the two groups.

Variable	Endoscopic group	Open group	Z value	*P*
Intraoperative time (min)	104 (85, 119.50)	72 (60, 85)	-7.22	<0.001
Intraoperative blood loss (ml)	20 (15, 30)	20 (10, 22.50)	-2.75	0.06
Number of central lymph nodes dissection (n)	8 (7, 9)	8 (7, 9)	-0.73	0.47
Postoperative 24-hour drainage volume (ml)	29.50 (21, 33.25)	20 (14, 26)	-4.06	<0.001
Indwelling time of drainage tubes (days)	3 (3, 3)	3 (3, 3)	-0.37	0.71
Length of hospital stay (days)	6.50 (6, 8)	6 (5, 7)	-1.16	0.25
Surgical costs (yuan)	3591 (3323.25, 3926.25)	3465 (3213.50, 3894.25)	-1.27	0.20

Values are Median (IQR).

### 3.3 Postoperative complications, neck discomfort, and satisfaction

No patients in either group experienced postoperative bleeding, infection, chylous leakage, or hypoparathyroidism. One patient in the endoscopic group developed temporary recurrent RLN injury, while two patients in the open group experienced the same. There were no permanent RLN injuries in the endoscopic group, whereas one patient in the open group developed a permanent RLN injury due to tumor invasion enveloping the RLN. The incidence of postoperative complications did not differ significantly between the two groups (*p*>0.05). The proportion of patients reporting neck discomfort was considerably lower in the endoscopic group compared to the open group (p<0.05). Three months postoperatively, patients in the endoscopic group expressed higher satisfaction with their incision than those in the open group (*p*<0.05) (**Table 3**).

**Table 3:** Postoperative complications, neck discomfort, and satisfaction in two groups.

Variable	Endoscopic group	Open group	Test Statistic	*P*
Bleeding	0	0	–	–
Infection	0	0	–	–
Chylous leakage	0	0	–	–
Hypoparathyroidism	0	0	–	–
Temporary RLN injury	1 (1.4)	2 (2.9)	–	1.0
Permanent RLN injury	0	1 (1.4)	–	1.0
Neck discomfort	8 (11.4)	25 (35.7)	Z=11.46	<0.001
Incision satisfaction				
Very satisfied	38 (54.3)	17 (24.3)	χ2 = 13.76	0.001
Satisfied	28 (40.0)	43 (61.4)		
Fair	4 (5.7)	10 (14.3)		
Dissatisfied	0	0		

Values are n (%).

### 3.4 Multivariate analysis

The two patient groups with different surgical approaches showed significant differences in age and gender. To exclude the influence of age and gender on surgical-related outcomes, multivariate regression analysis was performed. The results indicated that both surgical approach (*p*<0.001) and age (*p*=0.02) were associated with operative time, while gender was not related to operative time. Only the surgical approach was significantly correlated with postoperative 24-hour drainage volume (*p*=0.003), neck discomfort (*p*=0.007), and incision satisfaction (*p*=0.004) (**Tables 4**, **5**).

**Table 4:** Multivariate linear regression analysis on the association of surgical approach, age, and gender on operative time and postoperative 24-hour drainage volume.

Variable	Intraoperative time			Postoperative 24-hour drainage volume				
	B	SE	Beta	*P*	B	SE	Beta	*p*
Surgical approach (Endoscopic group vs Open group)	25.66	3.56	0.57	<0.001	7.38	2.40	0.30	0.003
Age	-0.40	0.17	-0.17	0.02	0.02	0.12	0.02	0.84
Gender (Male vs Female)	6.88	4.37	0.11	0.12	0.14	2.94	0.004	0.96

**Table 5:** Multivariate logistic regression analysis on the association of surgical approach, age, and gender on patients’ neck discomfort and satisfaction with the surgical incision.

Variable	Neck discomfort		Incision satisfaction	
	OR (95% CI)	*P*	OR (95% CI)	*p*
Surgical approach (Endoscopic group vs Open group)	4.16 (1.47-11.76)	0.007	3.24 (0.38-1.98)	0.004
Age	0.99 (0.94-1.04)	0.60	0.99 (-0.04-0.03)	0.79
Gender (Male vs Female)	0.46 (0.16-1.29)	0.14	0.88 (-1.09-0.84)	0.80

## 4 Discussion

Traditional open thyroid surgery is commonly used due to its simplicity and effectiveness; however, it leaves a visible neck scar, which can cause psychological distress for patients. The rapid advancement of endoscopic thyroid surgery has made it a popular option among patients and doctors due to its more concealed incisions, which more effectively address patients’ cosmetic concerns (8). However, various endoscopic approaches come with their advantages and challenges. The chest-breast approach requires a longer surgical path and a larger area of subcutaneous dissection, resulting in more hidden trauma (9). Transoral endoscopic thyroid surgery, classified as a clean-contaminated procedure, carries a risk of deep neck infections and mental nerve injury, necessitating prophylactic antibiotics (10). Moreover, most approaches involve the infusion of CO_2_ to establish an operating space, which may lead to complications such as CO_2_ embolism, pneumothorax, subcutaneous emphysema, and hypercapnia (11, 12). The gasless axillary approach is more technically challenging than the subclavian approach due to its longer surgical path and interference from the sternum and clavicle (13).

The gasless subclavian approach for endoscopic thyroid surgery eliminates the need for CO_2_ infusion, features a shorter surgical path, and enters through the natural space between the sternal and clavicular heads of the sternocleidomastoid muscle, resulting in less trauma and a concealed incision that aligns with patients’ cosmetic concerns. Our study revealed no significant differences between the subclavian endoscopic approach and traditional open thyroid surgery regarding the number of central lymph node dissections, intraoperative blood loss, drainage tube indwelling time, and postoperative complication rates (p>0.05). These findings suggest that the subclavian endoscopic approach provides comparable effectiveness and safety to traditional open surgery. In our study, one patient in the endoscopic group and two in the open group experienced temporary RLN injury, all of which recovered within three months postoperatively, likely due to intraoperative traction or edema. Only one patient (in the open group) developed permanent RLN paralysis due to tumor invasion of the RLN. Intraoperative nerve monitors effectively identified, exposed, and protected the RLN (14, 15). Gentle manipulation and avoiding traction on the nerve are crucial, and energy devices should be used at a safe distance to prevent thermal injury. None of the patients experienced postoperative bleeding, infection, chylous fistula, or hypoparathyroidism. Parathyroid glands should be preserved *in situ* during operation to prevent postoperative hypoparathyroidism. Using nanocarbon can assist in identifying the parathyroid glands (16), and parathyroid glands that are inadvertently resected or lack adequate blood supply should be promptly transplanted autologously.

Compared to traditional open thyroidectomy, the subclavian endoscopic approach considerably reduces postoperative neck discomfort (*p*<0.001), likely due to the preservation of the anterior cervical skin flap and fascia, maintaining the region’s anatomical integrity. Furthermore, the subclavian incision is well-concealed and easily covered by clothing, leading to higher patient satisfaction with the incision appearance (*p*<0.05). This approach does not prolong hospital stays or increase surgical costs (*p*>0.05), fulfilling cosmetic expectations without imposing additional financial burden.

This approach was frequently chosen by younger female patients, likely reflecting greater cosmetic concerns within this demographic. The endoscopic group exhibited longer operative times than the open group, possibly due to the need for subcutaneous tunnel creation and continuous adjustment of endoscopic retractors to maintain the operating space. The limited operative field and smoke generated by energy devices, such as ultrasonic scalpels, may further compromise endoscopic visibility and contribute to prolonged surgery duration. These factors collectively influence operative duration. Although the surgeon had prior endoscopic experience, some procedures in the endoscopic group were performed during the early phase of implementing gasless subclavian endoscopic thyroidectomy at our institution. This also increased the operation time of the endoscopic group to some extent. As the case volume increased, the surgical team became more proficient and coordinated, resulting in reduced operative times relative to the early phase. Further reductions in operative duration are anticipated with continued refinement and technical proficiency. The endoscopic group exhibited slightly higher 24-hour postoperative drainage than the open group, potentially due to a marginally extended skin flap dissection along the lateral neck and increased tissue trauma from the infraclavicular approach. Moreover, the open group benefited from direct visualization for precise vessel ligation. However, these factors did not prolong the drainage tube indwelling time.

The limitations of this study lie in relying solely on patients’ subjective perceptions to evaluate their satisfaction with postoperative incisions, lacking objective assessments. Additionally, the short follow-up period prevents the evaluation of the long-term outcomes of the subclavian endoscopic thyroidectomy. In future research, we will extend the follow-up duration and employ standardized scales to assess postoperative incision satisfaction and quality of life, further investigating the long-term efficacy of the subclavian endoscopic thyroidectomy.

In summary, subclavian endoscopic thyroidectomy offers a shorter surgical path and reduced trauma compared to other endoscopic approaches. Compared to traditional open surgery, it demonstrates comparable efficacy and safety, while significantly decreasing neck discomfort and meeting patients’ aesthetic expectations. It is economical, widely applicable, and merits broader clinical adoption. Although associated with slightly longer operative times and increased drainage, and mostly used for unilateral thyroid cancer treatment due to tracheal obstruction. These challenges are expected to diminish with ongoing surgical improvements.

## Data Availability

The raw data supporting the conclusions of this article will be made available by the authors, without undue reservation.
