# Supplementary material for: The efficacy of gasless endoscopic thyroidectomy via trans-subclavian approach in unilateral papillary thyroid carcinoma
Source: Front Endocrinol (Lausanne). 2025 Aug 27;16:1621481. doi: 10.3389/fendo.2025.1621481 (PMC12420322; doi:10.3389/fendo.2025.1621481)
Supplement: Supplementary file 1 [file Table1.docx]

Supplementary table Comparison of operative times between the mature-phase endoscopic group (excluding early-phase cases) and the open group

|  | Mature-phase endoscopic group (n=37) | Open group (n=70) | Test value | *P* |
| --- | --- | --- | --- | --- |
| Intraoperative time (min), median (IQR) | 99 (85, 115.50) | 72 (60, 85) | Z=-5.64 | ＜0.001 |
